# The evolving role of non-invasive assessment for liver fibrosis

**DOI:** 10.1093/gastro/goae080

**Published:** 2024-08-22

**Authors:** Peng Wang, Calvin Q Pan, Yali Liu, Jing Zhang

**Affiliations:** The Third Unit, Department of Hepatology, Beijing Youan Hospital, Capital Medical University, Beijing, P. R. China; Division of Gastroenterology, Department of Medicine, NYU Langone Medical Center, New York University School of Medicine, New York, NY, USA; The Third Unit, Department of Hepatology, Beijing Youan Hospital, Capital Medical University, Beijing, P. R. China; The Third Unit, Department of Hepatology, Beijing Youan Hospital, Capital Medical University, Beijing, P. R. China

Liver pathology remains the gold standard for diagnosing liver fibrosis, yet it falls short in meeting the broad clinical needs for screening, diagnosis, monitoring, and efficacy assessment. Non-invasive tests (NITs) offer a crucial alternative. The recent review by Lai *et al.* comprehensively covers various NITs and their applications across liver diseases of different etiologies, enhancing their clinical use by healthcare providers [1]. This commentary expands on the evolving role of non-invasive assessment for liver fibrosis discussed in the review by Lai *et al.*, highlighting recent advancements and comparisons, and the significance of dynamic changes in the research development and clinical utilization of non-invasive assessment in patients with liver disease.

## Challenges in the assessment of liver fibrosis

As chronic hepatitis B is declining and hepatitis C is approaching elimination, the clinical utilization of NITs has increased for metabolic dysfunction-associated steatotic liver disease (MASLD), formerly known as non-alcoholic fatty liver disease (NAFLD). With the Food and Drug Administration (FDA)’s approval of resmetirom for non-cirrhotic metabolic dysfunction-associated steatohepatitis (MASH), NITs have become essential for both diagnosing liver fibrosis and evaluating efficacy after intervention or monitoring for disease progression in untreated individuals, bypassing the need for pathology. Despite the availability of the enhanced liver fibrosis (ELF) test, which remains the only FDA-approved NIT, evidence supporting its post-intervention use is insufficient. Given the prevalence of MASLD, screening high-risk populations and managing fibrosis through stratified approaches rely heavily on NITs.

## Comparison of NITs and NIT-assisted patient screening

Direct comparisons of NITs reveal variability due to cohort differences, such as MASH prevalence and fibrosis composition. Recent studies have conducted head-to-head comparisons to identify the most effective NITs. Kim *et al.* found that MEFIB (magnetic resonance elastography plus fibrosis-4 [FIB-4]) outperformed MAST (magnetic resonance imaging [MRI]-aspartate aminotransferase [AST]) and FAST (FibroScan-AST) in detecting significant fibrosis [2]. MEFIB had higher positive and negative predictive values, indicating a smaller gray area. A study analysing 966 blood specimens from liver biopsy patients compared several markers and found that SomaSignal; age, diabetes, PRO-C3, and platelets panel (ADAPT); and liver stiffness measurement (LSM) had area under the curves (AUCs) of >0.8 for detecting advanced fibrosis [3]. The best markers for screening at-risk nonalcoholic steatohepatitis (NASH) included SomaSignal, ADAPT, MACK-3, and Pro-C3. In another large cohort study with 1,097 individuals, only NIS-4 was superior to FIB-4 in predicting at-risk NASH, with an AUC of 0.81 [4]. ELF, PROC3, and FibroMeter vibration-controlled transient elastography (VCTE) outperformed FIB-4 in predicting various stages of fibrosis. The SAFE score, released in 2023, showed superior performance over FIB-4 and NAFLD fibrosis scores (NFS) in distinguishing between F0/1 and ≥F2 [5]. Although the latest hepatitis B virus guidelines 2024 set APRI 0.5 and LSM 7.0 kPa as the cut-off for F2 fibrosis, the prediction of F2 and at-risk NASH is both critical and challenging, especially given the high screening failure rates in drug trials. Many manufacturers now include NITs in screening processes to reduce these rates. Therefore, accurate diagnosis of F2 fibrosis and at-risk NASH remains a pressing need.

## The role of NITs in monitoring patients with MASLD

NITs can predict the long-term prognoses of MASLD patients. However, liver fibrosis is dynamic, necessitating repeated testing for accurate monitoring. A longitudinal study of 44,481 adults with obesity and/or type 2 diabetes and an FIB-4 score of ≥1 showed that changes in FIB-4 scores over 12 months were associated with liver event incidence over 10 years [6]. In a veteran database of 202,319 patients, persistently high FIB-4 scores were linked to higher risks of hepatocellular carcinoma (HCC) and cirrhosis [7]. Changes in VCTE and NFS also predict liver disease progression and clinical events, unlike FIB-4 and ELF.

## NITs for the assessment of treatment response

Evaluating liver histologic response using NITs post-treatment presents challenges. Different drugs and interventions produce varied histologic recovery characteristics. The FLINT trial showed that reductions in APRI, FIB-4, and NFS at 72 weeks were linked to fibrosis improvement, with early reductions predicting fibrosis reversal [8]. In a phase 3 trial, significant proton density fat fraction reduction was associated with improved NASH and liver fibrosis outcomes [9]. These studies suggest a need for further research to clarify the concordance between NIT changes and liver histology post-treatment.

## NITs in general population screening

Screening for fatty liver and fibrosis staging in the community is critical for stratified management. FIB-4 is widely recommended, but its limitations include false positives and negatives. A study across five regions found significant false-negative and overdiagnosis rates for FIB-4 and NFS, particularly in at-risk populations [10]. Combining FIB-4 with VCTE improves diagnostic accuracy, reducing the need for biopsies. The ADAPT score, which is superior to LSM for excluding advanced fibrosis, offers a promising two-step screening approach. The SAFE model showed higher accuracy than FIB-4 and NFS in distinguishing between early and advanced fibrosis stages, validated in multiple cohorts [5].

## Further investigations on cost-effectiveness

Assessing the economic impact of different NITs can help healthcare systems to allocate resources more efficiently, ensuring that patients receive the most effective and affordable care. Cost-effectiveness studies should consider not only the direct costs of the tests, but also the long-term savings from early detection and intervention, which can prevent costly complications and advanced liver disease. Integrating cost-effectiveness analysis with clinical performance data will provide a comprehensive framework for the optimal use of NITs in managing liver fibrosis.

## Summary

While existing NITs provide valuable tools for liver fibrosis assessment, there remains a critical need for improved methods to enhance accuracy and applicability, particularly in post-intervention settings and community screenings. The proposed NITs for screening at-risk patients and monitoring those who have MASLD are summarized in Table 1. Further cost-effectiveness studies will play a crucial role in guiding clinicians and healthcare providers when making informed decisions about the use of NITs, ultimately improving patient outcomes and resource utilization in the management of liver fibrosis.

**Table 1.** Proposal for screening at-risk patients and monitoring those who have MAFLD using non-invasive testing

Proposal for screening patients at risk for MAFLD using non-invasive testing								
NITs	Applications	Population	Tools	Risk stratification	Predictive efficacy	AUC	Comparison	Weaknesses
FIB-4	Exclusion of F3 fibrosis	5,129 cases from five cohorts (multicenter) (PMID: 34971806)	Serological	LSM = 8 or 12 kPa	Sensitivity: 37%	NA	NA	Easily missed in at-risk individuals
					Specificity: 69%			High false-positive rate
NFS			Serological	LSM = 8 or 12 kPa	Sensitivity: 52%	NA	Superior to FIB-4 in general population	Influenced by LSM
					Specificity: 69%			High false-positive rate
FIB-4 + VCTE	Exclusion of advanced fibrosis	5,735 patients from 37 studies (meta-analysis) (PMCID: PMC8995830)	Serological	FIB-4<1.3 or ≥2.67, VCTE <8.0 or ≥10.0 kPa	Sensitivity: 38%	0.85	Superior to FIB-4, NFS	NA
			+Radiographic		Specificity: 90%			
ADAPT	Exclusion and inclusion of F3–F4 fibrosis	713 cases from general population (PMID: 33252454)	Serological	ADAPT = 4.45 or 7.15	PPV: 92.5%	0.88	Superior to APRI, FIB-4, BARD, NFS	NA
					NPV: 98%			
SAFE	Exclusion and prediction of F2 fibrosis	11,953 cases from NHANES III (PMCID: PMC9613815)	Serological	SAFE = 0 or 100	NPV: 92%	>0.80	Superior to FIB-4, NFS	High false positives in older age groups

**Proposal for monitoring patients with diagnosis of MAFLD using non-invasive testing**								

**NITs**				**Outcomes**				

FIB-4				Liver-related events; cirrhosis, HCC; severe liver disease				
VCTE				Cirrhosis progress				
ELF, VCTE				Liver-related events				
LSM				Liver-related events and death				

ADAPT = age, diabetes, PRO-C3, and platelets panel, ELF = enhanced liver fibrosis, LSM = liver stiffness measurement, MAFLD = metabolic dysfunction-associated steatotic liver disease, FIB-4 = fibrosis-4, NA = not application, NFS = NAFLD fibrosis scores, NITs = non-invasive testing, SAFE = steatosis-associated fibrosis estimator, VCTE = vibration-controlled transient elastography.
